# Catalytic Thr or Ser Residue Modulates Structural Switches in 2-Cys Peroxiredoxin by Distinct Mechanisms

**DOI:** 10.1038/srep33133

**Published:** 2016-09-15

**Authors:** Carlos A. Tairum, Melina Cardoso Santos, Carlos A. Breyer, R. Ryan Geyer, Cecilia J. Nieves, Stephanie Portillo-Ledesma, Gerardo Ferrer-Sueta, José Carlos Toledo, Marcos H. Toyama, Ohara Augusto, Luis E. S. Netto, Marcos A. de Oliveira

**Affiliations:** 1 Instituto de Biociências, Campus do Litoral Paulista, Universidade Estadual Paulista Júlio de Mesquita Filho, São Vicente, São Paulo, 11330-900, Brazil; 2 Departamento de Bioquímica, Instituto de Química, Universidade de São Paulo, São Paulo, 05508-090, Brazil; 3 Facultad de Ciencias – Universidad de la República – Montevideo, Uruguay; 4 Departamento de Química, Faculdade de Filosofia, Ciências e Letras de Ribeirão Preto, Universidade de São Paulo, Ribeirão Preto – SP, 14040-901, Brazil; 5 Departamento de Genética e Biologia Evolutiva, Instituto de Biociências, Universidade de São Paulo, São Paulo, 05508-090, Brazil

## Abstract

Typical 2-Cys Peroxiredoxins (2-Cys Prxs) reduce hydroperoxides with extraordinary rates due to an active site composed of a catalytic triad, containing a peroxidatic cysteine (C_P_), an Arg, and a Thr (or Ser). 2-Cys Prx are involved in processes such as cancer; neurodegeneration and host-pathogen interactions. During catalysis, 2-Cys Prxs switch between decamers and dimers. Analysis of 2-Cys Prx structures in the fully folded (but not locally unfolded) form revealed a highly conserved, non-conventional hydrogen bond (CH-π) between the catalytic triad Thr of a dimer with an aromatic residue of an adjacent dimer. In contrast, structures of 2-Cys Prxs with a Ser in place of the Thr do not display this CH-π bond. Chromatographic and structural data indicate that the Thr (but not Ser) destabilizes the decamer structure in the oxidized state probably through steric hindrance. As a general trend, mutations in a yeast 2-Cys Prx (Tsa1) favoring the dimeric state also displayed a decreased catalytic activity. Remarkably, yeast naturally contains Thr-Ser variants (Tsa1 and Tsa2, respectively) with distinct oligomeric stabilities in their disulfide states.

Typical 2-Cys peroxiredoxins (2-Cys Prx) from the Prx1/AhpC sub-group comprise a large class of thiol-specific antioxidant enzymes capable of reducing hydroperoxides with high efficiency (*k* = 10^6^–10^8^ M^−1^ s^−1^) and specificity^1^,^2^,^3^,^4^,^5^. The reactivity of 2-Cys Prx toward peroxides is considered extraordinary as they react one to ten million times faster than free Cys^6^. Due to their catalytic properties these enzymes are considered H_2_O_2_ sensors, which is probably related with the association of 2-Cys Prx with processes such as tumor suppression, neuronal differentiation and cardiovascular disease (reviewed in refs 7,8). 2-Cys Prxs make use of a fully conserved Cys residue, known as the peroxidatic cysteine (C_P_), for the reduction of hydroperoxides, which results in the formation of a sulfenic acid (C_P_-SOH) (Fig. 1a,b). The basic unit of 2-Cys Prxs is a homodimer (α_2_) and during the catalytic cycle a second Cys residue, the so-called resolving cysteine (C_R_) reacts with C_P_-SOH of the adjacent monomer (Fig. 1b,c) to form an intermolecular disulfide bond (reviewed in refs 8 and 9). A partial unfolding of the α-helix containing C_P_ is required for the intermolecular disulfide formation and thioredoxin (Trx) is commonly the electron donor to reduce the disulfide^7^,^8^,^9^ (Fig. 1a–c). Therefore, during catalysis, 2-Cys Prx enzymes assume two structural states: fully folded (FF) and locally unfolded (LU). Alternatively, C_P_-SOH can further react with additional hydroperoxide molecules giving rise to hyperoxidized states such as sulfinic (C_P_-SO_2_H^−^) and sulfonic (C_P_-SO_3_H^−^) acids (Fig. 1b–d). These hyperoxidized forms cannot be reduced by Trx, however in eukaryotic organisms C_P_-SO_2_H can be reduced back to C_P_-SOH by an ATP dependent sulfiredoxin^8^.

In the FF state, C_P_ is located in the first turn of an α-helix, surrounded by the Thr (or Ser, in some cases) and the Arg^9^,^10^. These three residues compose the catalytic triad, which has been implicated in the reactivity and specificity of Prx towards hydroperoxides^8^,^9^,^10^. Furthermore, transient interactions in the active site of Prxs are thought to play a crucial role in the stabilization of the transition state formed during the S_N_2 reaction^11^,^12^. There is evidence that the Arg residue is indispensable for Prx activity, since the substitution of the residue profoundly affects C_P_ reactivity^13^,^14^,^15^,^16^,^17^. In contrast, studies involving the substitution of the conserved Thr (or Ser) residue are scarcer and show less drastic effects on catalysis^13^,^17^.

Another intriguing feature of 2-Cys Prx enzymes is their ability to switch among distinct quaternary structures, which is affected by redox state, among other factors^8^,^9^,^18^,^19^,^20^,^21^. These enzymes are α_2_ homodimers that can associate in (α_2_)_5_ decamers, *i.e.* pentamers of dimers. The factors governing the stabilities and the possible significance of these distinct quaternary structures are not completely understood, yet the formation of an intermolecular disulfide bond is known to destabilize the decamer (Fig. 1b–e). In the case of a 2-Cys Prx from *Salmonella typhimurium* (StAhpC), decamer stability was associated with an increase in the peroxidase activity^4^. Like the reduced enzymes, hyperoxidized 2-Cys Prxs appear preferentially as decamers (or even higher molecular weight complexes), whereas the presence of intermolecular disulfides destabilizes decamers into dimers^4^,^20^,^21^,^22^,^23^,^24^,^25^ (Fig. 1). Besides redox state, several post-translational modifications also affect the stabilities of 2-Cys Prx quaternary structures, which impact their activities^26^,^27^,^28^.

2-Cys Prxs play central roles in redox signaling but mechanisms governing their structural transitions are poorly understood. In this work, all analyzed 2-Cys Prx structures in the FF state (but not LU state) showed that the catalytic Thr (Thr44 in Tsa1, a 2-Cys Prx from *S. cerevisiae*) is located at the interface between two dimers, making a non-conventional hydrogen bond (H bond) with the π system of a Tyr/Phe residue (Tyr77 in Tsa1) of the adjacent dimer. In contrast, 2-Cys Prxs with Ser in place of catalytic Thr (Ser45 in Tsa2 from *S. cerevisiae*) always appear as decamers and do not present any CH-π bond. Chromatographic and structural data indicate that the Thr (but not Ser) destabilizes the decamer structure during disulfide formation (FF to LU transition) probably through steric hindrance, which affect the ability of 2-Cys Prx to reduce hydroperoxides.

## Results

### Structural characterization of typical 2-Cys Prx enzymes

The reversible transition between the dimeric and decameric states is a remarkable feature of 2-Cys Prxs^4^,^18^,^19^,^20^,^21^,^22^,^23^,^24^,^25^,^26^,^27^,^28^,^29^,^30^. Since the interactions between dimers are relevant for decamer stabilization, we analyzed the structures of 2-Cys Prx enzymes available in *Protein Data Bank*. The analysis of the yeast Tsa1 crystallographic structure^31^ in the reduced form revealed that (α_2_)_5_ decamer is stabilized by several non-covalent interactions at the dimer-dimer interface (Fig. 2a–c). Among these interactions, the Cγ atom of Thr44 acts as H-bond donor to the π-system of Tyr77 in the adjacent dimer (Fig. 2d). This CH-π bond is a non-conventional H bond that plays important contributions in protein structure and function (reviewed in refs 32,33). In the Tsa1 decameric structure, all five dimer-dimer interfaces have two CH-π H bonds between Thr44 and Tyr77 residues (Fig. 2e), and involves residues that are part of the so-called region I (Thr44) and region II (Tyr77), which are complementary at the dimer-dimer interface^21^.

Remarkably, the CH-π H bonds between the catalytic Thr and the Tyr of the adjacent dimer are fully conserved among all analyzed structures of 2-Cys Prx (Prx1/AhpC) in the FF state (See Supplementary Fig. 1 and Fig. 2d and Supplementary Table 1). In some cases, the Tyr is substituted by a Phe residue, which can also accept H bonds through electrons of its π-system (See Supplementary Fig. 1 and Supplementary Fig. 2a,c,d).

In the LU state, the orientation between Thr44 and Tyr77 residues changes, and consequently the CH-π H bond is disrupted (Fig. 2e,f; See Supplementary Fig. 2b). As the microenvironment around the active site Thr is highly crowded (See Supplementary Fig. 3a,b,d–g), structural rearrangements of the neighboring residues during FF → LU transition are likely required to allow Thr44 to swing (Fig. 2e,f), probably provoking disruption of other non-covalent interactions at the dimer-dimer interface.

In contrast, the microenvironment around the active site Ser (equivalent to Thr44 in Tsa1) in AhpC from *Amphibacillus xylanus* (See Supplementary Fig. 3c) and in Tsa2 from *Saccharomyces cerevisiae* (See Supplementary Fig. 3h–k) is less crowded. Ser45 in the decameric structure of Tsa2 (which shares 86% of identity with Tsa1) appears as distinct rotamers in distinct chains, indicating high flexibility of this residue^34^. Remarkably, the CH-π hydrogen bond was not observed between Ser45 and Tyr78 (Tyr77 in Tsa1) in any of the Ser45 rotamers in Tsa2. Additionally, some of the chains from the Tsa2 decamer are in the FF state, while others are in the LU state. In fact, analysis of B factors in the α-helix containing the C_P_ of the decameric Tsa1 and Tsa2 structures revealed that the thermal variation of Ser45 in Tsa2 is remarkably higher than the corresponding value for Thr44 in Tsa1 (Fig. 2g,h).

Therefore, it is possible that the conserved Thr/Ser not only participates in catalysis^10^,^12^, but is also involved in modulating the oligomeric state of the enzyme. For instance, while Thr44 Cγ atom of Tsa1 interacts with the neighboring dimer, the Oγ atom has a polar interaction with the Sγ atom of C_P,_ which is further stabilized by polar contacts with the guanidine group of Arg123 (Fig. 2d,e; See Supplementary Fig. 2d). Furthermore, the catalytic triad of 2-Cys Prx is located near the dimer-dimer interface delimited by Tyr77 (Fig. 2d). Taken together, Thr44 provides a physical interaction between the catalytic center and the dimer-dimer interface, and indicates that the presence of Thr or Ser in the catalytic triad can influence the equilibrium among 2-Cys Prx quaternary structures.

### Structural characterization of Tsa1 Thr44 mutants

Based on the structural considerations described above, we hypothesized that substitution of Thr44 in Tsa1 by other residues would affect decamer stability. Since the theoretical molecular weight of Tsa1 homodimers (~48 kDa) in solution is easily distinguishable from Tsa1 decamers (~240 kDa), we employed size exclusion chromatography (SEC) to evaluate the effects of Thr44 substitutions on Tsa1 quaternary structure. Under reducing conditions, Tsa1^WT^, Tsa1^T44A^, and Tsa1^T44S^ eluted as decamers (Fig. 3a–c, solid lines), whereas Tsa1^T44V^ eluted as a mixture of dimers and decamers, with the dimeric forms being clearly more predominant (Fig. 3d, solid line). As expected, the oxidized (disulfide) samples favored dissociation of decamers^21^ as compared with the reduced forms (Fig. 3, dashed lines). The exception was Tsa1^T44S^, which migrated exclusively as decamers in both the oxidized and reduced forms (Fig. 3c). In contrast, oxidized Tsa1^T44V^ migrated mainly as dimer (Fig. 3d, dashed line). With intermediate behavior, comparable amounts of dimers and decamers of Tsa1^WT^ and Tsa1^T44A^ were detected in oxidizing conditions (Fig. 3a,b, dashed lines). In summary, two of the Thr mutations provoked dramatic effects on the quaternary structure, with Tsa1^T44S^ favoring decamer stabilization and Tsa1^T44V^ favoring dimer stabilization.

### Kinetic characterization of Tsa1 Thr44 mutants

Next, we examined the effects of the mutations on Tsa1 reactivity towards hydroperoxides using the Trx system coupled assay. Tsa1^WT^ and Tsa1^T44S^ presented catalytic efficiencies towards H_2_O_2_ in the 10^4^ M^−1^ s^−1^ range (See Supplementary Fig. 5a,c), similar to the previously obtained value^35^. On the other hand, Tsa1^T44A^ presented a slight decrease in activity (See Supplementary Fig. 5b), whereas the catalytic efficiency of Tsa1^T44V^ for H_2_O_2_ decreased by two orders of magnitude and the K_M_ value (520 μM) increased by two orders of magnitude (See Supplementary Fig. 5d). All enzymatic parameters are summarized in Supplementary Table 2. The results for Tsa1^T44V^ are consistent with a previous study, which showed that the catalytic efficiency of human PrxV, with a Thr for Val mutation, also decreased the catalytic efficiency by a factor of one thousand^13^. All kinetic parameters were determined under conditions where Trx (at 2 μM) was not limiting the catalysis (See Supplementary Fig. 5e).

In the case of cumene hydroperoxide (CHP) reduction, Tsa1^WT^ and Tsa1^T44S^ also presented similar enzymatic parameters (See Supplementary Fig. 5a–c, insets). In contrast to H_2_O_2_ reduction, Tsa1^T44A^ presented a very high K_M_ (300 μM) for CHP and consequently a four-fold decrease in the catalytic efficiency (See Supplementary Fig. 5b, inset). In the case of the Tsa1^T44V^ mutant, an even more drastic drop in the reduction of CHP was observed (See Supplementary Fig. 5d, inset), precluding the determination of the kinetic parameters (Supplementary Table 2). Similarly, a 2-Cys Prx from *L. donovani* (TXNPx) also displayed negligible peroxidase activity towards organic peroxides when the homologous Thr residue was mutated to Val (TXNPx^T49V^)^13^. These results indicate that Val mutations are not well tolerated at this position, and that eliminating the hydroxyl group from the side chain impairs the catalytic activity of 2-Cys Prxs. The factors underlying why organic peroxide reduction is more inhibited than H_2_O_2_ reduction are not clear, and may be related to distinct effects of amino acid side chains on stabilization of the respective transition states with H_2_O_2_ and CHP; and/or with the release of the corresponding leaving groups.

We also evaluated the effects of the Thr mutations by non-reducing SDS-PAGE. Reduced proteins were oxidized with three equivalents of H_2_O_2_ or CHP and the disappearance of monomers was monitored. Among the three mutants, only Tsa1^T44V^ presented a significant delay in disulfide formation for both H_2_O_2_ and CHP treatments (See Supplementary Fig. 6). For oxidations of 2-Cys Prx by H_2_O_2_, a similar trend in the reactivities as those observed in the Trx system coupled assay: Tsa1^WT^ ~ Tsa1^T44S^ > Tsa1^T44A^ ≫ Tsa1^T44V^, taking into account that the SDS PAGE assay has a low resolution in time, being unable to discriminate moderate from very fast rates. For oxidation by CHP, similar tendency was also observed, but Tsa1^T44V^ was oxidized in seconds (See Supplementary Fig. 6), whereas in the Trx system coupled assay, no NADPH consumption was detected (See Supplementary Fig. 5d). These apparent inconsistences are possibly related with differences in the principles underlying these two assays.

**Figure 1:**
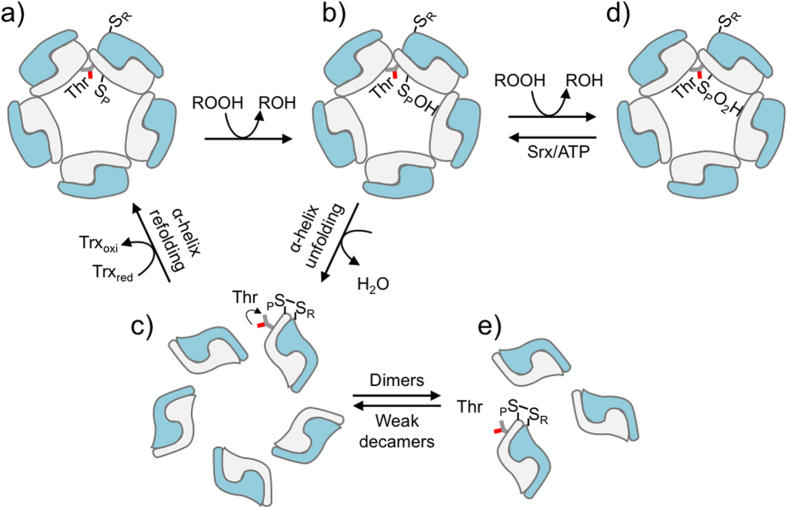
Proposed mechanism for hydroperoxide reduction by 2-CysPrx (Ahpc-Prx1). (**a**) C_P_ in the fully folded (FF) form (S^−^_p_) displays high reactivity towards hydroperoxides and stabilizes the decamer state. This quaternary structure corresponds to a strong decamer. (**b**) The oxidation product (sulfenic acid - S_p_OH), also a strong decamer, and can have two outcomes: condensation or hyperoxidation. (**c**) The intermolecular disulfide is formed after a local unfolding process, because the two Cys residues are far away in the FF state, which destabilizes the decamer. In this work, we show that the extra Cγ in the side chain of catalytic Thr (Thr 44 in Tsa1) contributes to decamer destabilization. The reduced form of typical 2-Cys Prx, which favors the strong decamer, is regenerated by Trx. The disulfide form of 2-Cys Prx can switch from a weak metastable decamer (**c**) to dimers (**e**). Alternatively, the sulfenic acid of 2-Cys Prx (**b**) can be hyperoxidized (**d**) by further oxidation of C_P_ to S_p_O_2_H by a second molecule of hydroperoxide. This process can be reversed by Srx, with ATP consumption.

In the case of the Trx system coupled assay, a complete turnover of the enzyme is required for NADPH consumption. Mutations affecting any of the three reactions (hydroperoxide reduction; condensation; disulfide reduction) or structural transitions (described in Fig. 1) might impair or even block the turnover of the enzyme. In contrast, only the oxidation and condensation reactions are measured in the non-reducing SDS-PAGE assay.

### Determination of apparent second order rate constants for the reaction between Tsa1 proteins and hydroperoxides

The interpretation of the results obtained by the Trx system - coupled assay is not always straightforward. Among other reasons this is complicated by the facts that the overall assay requires three different enzymes, involving various consecutive thiol-disulfide exchange reactions (among 2-Cys Prx, Trx and Trx reductase 1) and that catalytic cycle of 2-Cys Prx enzymes itself is complex, comprising three reactions (mentioned above) and conformational changes (LU-FF transitions; dimer–decamer switches)^9^. Therefore, direct rate constants between hydroperoxides and reduced Tsa1 proteins were determined by taking advantage of the changes in their intrinsic fluorescence upon oxidation (Fig. 4a). Under pseudo first-order conditions (excess peroxide), a two phase kinetic profile was observed for Tsa1^WT^ (Fig. 4a,b). The first rapid phase is accompanied by a drop in fluorescence (first 5 ms), after an apparent 2 ms lag time, which coincides with the dead time of the instrument. The second phase is slower and is accompanied by a fluorescence increase.

Recently, a similar kinetic profile was described for a bacterial 2-Cys Prx (StAhpC)^36^. In order to fit the experimental data, the authors proposed a three phase kinetic model. In the first step, H_2_O_2_ would reversibly bind to the StAhpC catalytic site, forming an enzyme - substrate complex, followed by C_P_ peroxidation (which was associated to the fast fluorescence decay) and resolution (which was associated to second fluorescence increase phase). However, in the case of Tsa1 and under our experimental conditions the rise in fluorescence (second slower phase) could not be satisfactorily fitted and therefore was not further analyzed here. Therefore, Tsa1 and StAhpC display different fluorimetric properties, which might be due to distinct structural features of Trp residues (See Supplementary Fig. 7) or due to the fact that Tsa1 is sensitive to hyperoxidation, whereas StAhpC is resistant^37^.

Nevertheless, the rate of the initial rapid fluorescence decay increased linearly with peroxide concentrations (Fig. 4b,c), indicating that this phase is associated with C_P_ oxidation. To test this assumption, we determined what would be the rate constant of C_P_ oxidation under pseudo first-order conditions, fitting the fast decay in the fluorescence to a single first-order exponential equation or the whole biphasic time course to a two-exponential function (two consecutive first-order processes) for Tsa1^WT^. Using either approach, the rate constant for the Tsa1^WT^ oxidation by H_2_O_2_ was close to 5 × 10^7^ M^−1^ s^−1^ (Fig. 4c). Overall, this fluorimetric approach yielded second order rate constants for the Tsa1^WT^ oxidation by H_2_O_2_ and CHP in the 10^6^–10^7^ M^−1^ s^−1^ range (Table 1), which agrees well with values previously determined by independent methods^3^. These convergences validated the fluorimetric method and made us confident to apply the same approach to the remaining Tsa1 mutants. Tsa1^T44S^ mutant was also oxidized by H_2_O_2_ and CHP with extraordinary rates but the fluorimetric changes during the reaction was distinct from Tsa1^WT^, showing only a fast fluorescence decay associated with C_P_ oxidation (See Supplementary Fig. 8). A decrease in reactivity was observed for both Tsa1^T44A^ and Tsa1^T44V^ enzymes (See Supplementary Fig. 8). Once again the Tsa1^T44V^ mutant was far less reactive (Table 1), while the Tsa1^T44A^ mutant was still quite active, one thousand fold faster than the free amino acid cysteine^6^, although at the same time, one thousand fold slower than the Tsa1^WT^. On the other hand, the corresponding rate constant for the oxidation of Tsa1^T44V^ could not be determined due to its very low reactivity (Supplementary Fig. 8). The fluorimetric traces for Tsa1^T44V^ correlated quite well with the non-reducing SDS-PAGE assay (See Supplementary Fig. 6), but not with the Trx system coupled assay possibly by reasons related to the distinct principles of these three assays, as discussed above.

It is worth mentioning that the values obtained for catalytic efficiencies in steady state kinetics (*k*_*cat*_/K_M_) were consistently in the range of 10^3^–10^4^ M^−1^ s^−1^, thus typically a thousand fold lower, than that obtained using fluorescence spectroscopy. This is probably due to other reactions and/or structural rearrangements limiting the overall turnover under steady-state conditions as mentioned above. It is worth mentioning that reduction of yeast TrxR1 by Trx1 and Trx2 are in order of 10^7^ M^−1^s^−1 ^38, reinforcing the hypothesis that structural rearrangements limit the reaction velocity. Nevertheless, the same trend was observed regardless of the approach (Trx coupled; non-reducing SDS-PAGE or fluorimetric assay) employed. In the case of Tsa1^T44V^, this mutation decreased the ability of Tsa1 to reduce hydroperoxides by several orders of magnitude, whereas the Tsa1^T44A^ mutation induced a less pronounced effect. These results indicate the importance of the polar hydroxyl group in the catalytic mechanism.

### Relationship between quaternary structures and catalytic activity of 2-Cys Prx

The results presented so far indicated that distinct residues present at position 44 of Tsa1 play critical roles in both decamer stabilization and peroxidase activity. Yeast Tsa2 (86% of amino acid identity with Tsa1) was also analyzed, since this enzyme naturally presents a Ser in the position equivalent to Thr44 in Tsa1^3^. Similar to the Tsa1^T44S^ mutant (Fig. 3c), Tsa2 migrated exclusively as decamers independent of the enzyme redox state (Fig. 5a), further supporting the hypothesis that Ser in place of Thr stabilizes the decameric state of 2-Cys Prxs. In addition, Tsa2 reduced H_2_O_2_ and CHP with extraordinary rates (Fig. 5b, Table 1; see Supplementary Fig. 9a,b).

As mentioned previously, the aromatic residue, Tyr77, also participates in the non-conventional CH-π H bond with Thr44. Therefore, the Tsa1^Y77A^ mutant was also produced, overexpressed, and purified. This mutant protein always eluted as dimers (Fig. 5c) and the peroxidase activity of Tsa1^Y77A^ was greatly reduced (Fig. 5d; see Supplementary Fig. 9c,e). Tyr77 performs multiple interactions besides the non-conventional CH-π hydrogen bond with Thr44 in the dimer-dimer interface (See Supplementary Fig. 3d–g) and its replacement by alanine may disrupt various forces that stabilize the decamer. Substitution of Tyr77 residue that plays no role on transition state stabilization^11^,^12^, but that dramatically affected the ability of Tsa1 to reduce peroxides suggested that the decameric quaternary structure is important to activity.

Furthermore, Tsa1^S78D^ mutant was also generated based on the information that in StAhpC and in HsPrx4, similar substitutions provoked decamer destabilization^4^,^25^. Thr77 in StAhpC (Ser78 in Tsa1) is located in the decameric interface of the enzyme but does not have direct interactions with any of the active site residues. Substitution of Thr77 by Asp in StAhpC precluded decamer formation^4^. As predicted, Tsa1^S78D^ also eluted as dimers, independent of the redox state (Fig. 5e) and the peroxidase activity was only residual in the Trx system coupled assay (Fig. 5f; see Supplementary Fig. 9d,f). Actually, the Tsa1^S78D^ mutation resulted in a more pronounced loss of peroxidase activity than what was reported for StAhpC^T77D 4^.

As an independent and confirmatory assay, non-reducing SDS PAGE revealed that rates of disulfide formation in Tsa1^Y77A^ and Tsa1^S78D^ were extremely slow (See Supplementary Fig. 9g,h), precluding the determination of the rate constants values by available methods (Table 1). In contrast, Tsa2 displayed extraordinary second order rate constants for reduction of H_2_O_2_ and CHP (Table 1).

Since Thr44 provides a physical interaction between the catalytic center and the dimer-dimer interface it is intuitive a relationship between activity and oligomerization. However, the correlation between these two processes is not perfect, probably reflecting the fact that other factors interferes with the peroxidase activity and also with decamer stabilization. Nevertheless, if we analyze the more pronounced effects in the oligomeric state, such as Tsa2/Tsa1^T44S^ (decamers with high activity) versus Tsa1^T44V^/Tsa1^Y77A^/Tsa1^S78D^ (dimers with residual activity) the interplay between activity and oligomerization is clear (Fig. 5). These results further indicate a positive trend between decamer structure and peroxidase activity, although other factors such as pH and peroxiredoxin concentration also affect the enzymatic activity of 2-Cys Prx^8^,^9^,^18^,^19^,^20^,^21^,^22^,^23^,^24^,^25^,^26^,^27^,^28^,^29^,^30^.

## Discussion

The equilibrium between dimers and decamers for 2-Cys Prx is dependent on C_P_ redox state among other factors^8^,^9^,^18^,^19^,^20^,^21^,^22^,^23^,^24^,^25^,^26^,^27^,^28^,^29^,^30^, with oxidation to disulfides typically favoring dimers. Therefore, such a quaternary structure switch may be considered as part of the 2-Cys Prx catalytic cycle^9^ (Fig. 1). Here, we present evidences that the presence of a Thr or Ser in the catalytic triad (Thr44 in Tsa1; Ser45 in Tsa2) plays a critical structural role in the stabilization or destabilization of 2-Cys Prx decamers upon disulfide formation (Fig. 1; Supplementary Fig. 10). In proteins containing a Thr in the catalytic triad, oxidation leads to decamer dissociation into dimers^9^ (Fig. 1c–e), while with proteins containing a Ser, at the analogous position, such dissociation is not observed. This is an interesting structural finding by itself and may indicate different non-redundant or complementary enzyme functions.

**Figure 2:**
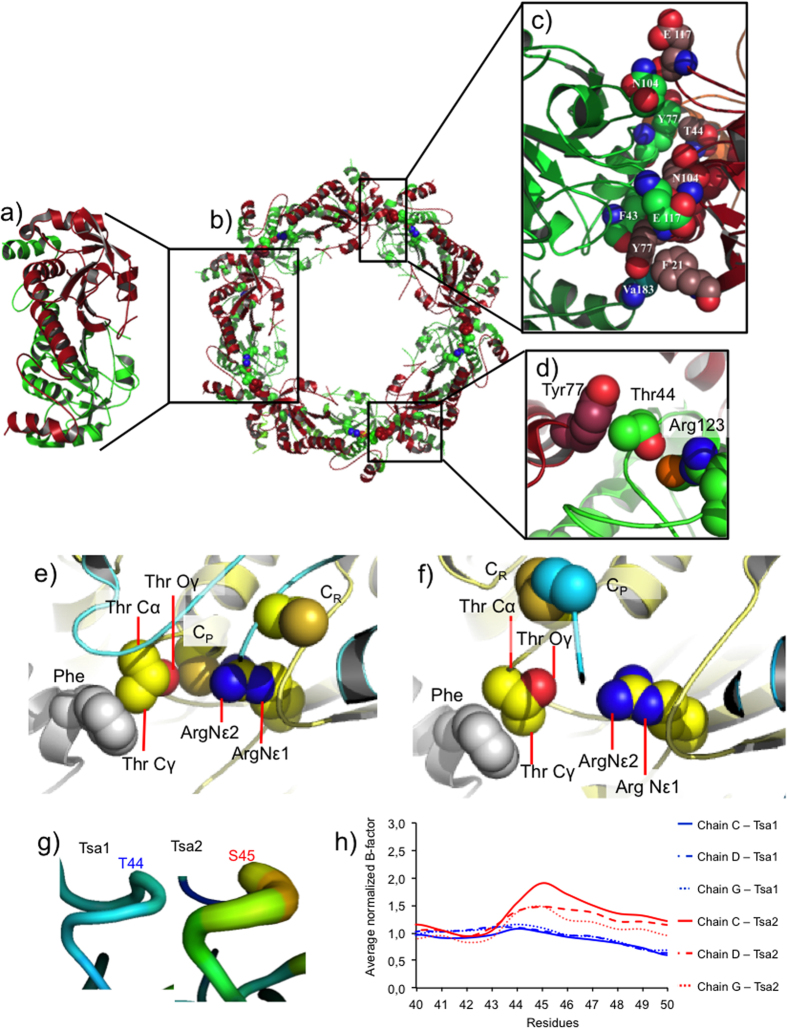
Structural features at the dimer-dimer interface. (**a–d**) Representations of Tsa1^C47S^ structure. (**a**) Tsa1 homodimer represented in cartoon with a chain colored in green and the other in red. (**b**) Tsa1 decamer as a pentamer of homodimers (pdb code = 3SBC). (**c**) Amino acid residues involved in the interactions between dimers are represented by spheres. (**d**) Thr44 Cγ perform a CH-π bond with the Tyr77 of the adjacent dimer, while the Oγ of Thr44 side chain is involved in polar interactions with C_P_ Sγ, which, in turn, is stabilized by the guanidine group of the Arg123. For (**c**,**d**), the carbon atoms are represented by colors similar to the polypeptide chain to which they belong, whereas N = blue, O = red and S = orange. For (**e**,**f**), the crystallographic structures of human Prx4 in the FF state ((**e**) 3TKP) and in the LU state ((**f**) 3TJB). The residues of catalytic triad and the conserved Tyr/Phe are represented by spheres and atoms are colored as follows: C = yellow, gray or light blue, according to the cartoon representation of the chain; N = blue; O = red; S = gold. (**g**) Wire representations of Thr/Ser region of yeast Tsa1 and Tsa2 (pdb codes 3SBC and 5DVB, respectively). The normalized B-factor was obtained by dividing the B-factor value of each Cα residue by the B-factor average value of all the Cα residues (normalized B-factor = B-factor of the residue/average B-factor of all residues). The wire thickness and color (blue = low to red = high) denotes the higher B factors for Tsa2 as compared to Tsa1. (**h**) Graphical detail of B-factor variation by amino acid in the portion containing the Thr or Ser from Tsa1 (blue curves) or Tsa2 (red curves), respectively. In the crystallographic structure of Tsa1, all chains are in the FF state while in the Tsa2 chains a mixture of FF, LU and intermediate states is found. Therefore, Tsa2 chains in FF state (C, D and G) and the respective chains of the Tsa1 were compared.

**Figure 3:**
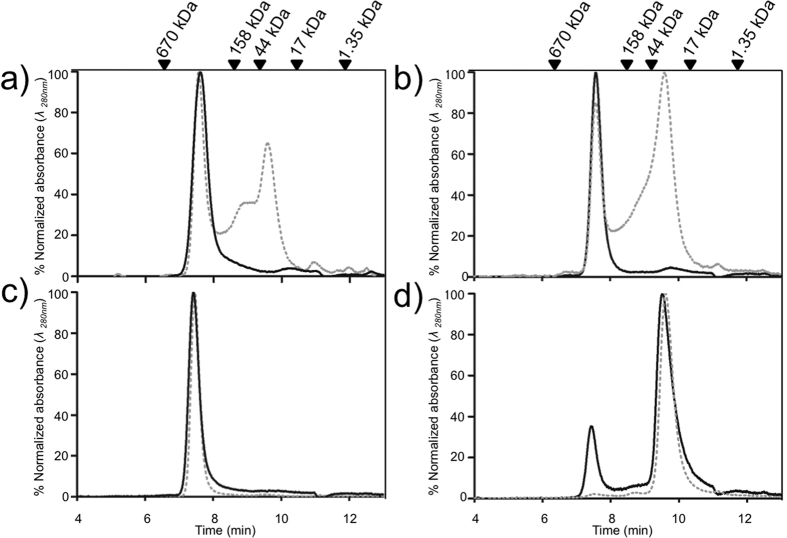
Size exclusion chromatography (SEC) profiles of Tsa1^WT^ and mutants in reduced and oxidized states. Samples were reduced with tris(2-carboxyethyl)phosphine (TCEP 5 mM; solid lines) or previously reduced with TCEP (5 mM) and then oxidized with H_2_O_2_ (1.2 molar equivalents; dashed lines) for 30 min at 25 °C. Tsa1^WT^ (**a**), Tsa1^T44A^ (**b**), Tsa1^T44S^ (**c**) and Tsa1^T44V^ (**d**). Enzyme (50 μM) was injected onto a HPLC system equipped with Phenomenex BioSep-SEC-S3000 column and resolved at a flow rate of 1 mL/min. The SEC column was calibrated with the following molecular weight standards: bovine thyroglobulin (670 kDa), bovine gamma globulin (158 kDa), ovalbumin (44 kDa), myoglobin (17 kDa), vitamin B_12_ (1.35 kDa) which are assigned at the top of the figure. The 100% value on the y-axis corresponds to the maximum absorbance detected in each chromatogram.

Through a comparative structural analysis of 2-Cys Prx enzymes, it became clear that although most residues in the dimer-dimer interface retain similar conformation between the FF and LU states, the conserved Thr residue (Thr44 in Tsa1) swings considerably (Fig. 2e,f; See Supplementary Fig. 2a,b). This reorientation was observed in all of the analyzed decamer structures and is associated with the disruption of the Thr-Tyr (or Phe) CH-π bond in the LU state. Moreover, we observed that the decameric structure was prevalent (compare Ser44 versus Thr44 and Ala44 versus Val44 in Fig. 3) for protein containing residues with smaller side chains, consistent with a steric factor underlying the decamer stability. In fact, the microenvironment around the active site Thr is more crowded (See Supplementary Fig. 3) and significant structural rearrangements of neighboring residues are likely required to allow Thr44 to swing during the FF-LU transition. Possibly, the extra Cγ in the Thr side chain (in comparison with Ser side chain) may create steric effects with nearby residues, thus disfavoring the decameric state during the transition from the FF to LU states. In fact, a weakly associated decamer (in the LU state) has been proposed as an intermediate between the FF decamer and the LU dimer^21^ (Fig. 1c) and other intermediate oligomeric structures have also been observed^14^,^19^. On the other hand, it is possible that smaller side chains of residues at position 44 (for example, the active site Ser in AhpC from *A. xylanus* and Tsa2 from *S. cerevisiae*, Supplementary Fig. 3) might allow for smoother FF–LU transitions, by avoiding major structural perturbations at the dimer-dimer interface.

Other mutations in the dimer-dimer interface (Thr77 residue of StAhpC and Thr118 in Prx4) were reported to provoke major changes in the stability of decamers^4^,^25^. However, crystallographic structures of these proteins did not reveal major alterations in the dimer-dimer interface. In most of these structures, the 2-Cys Prx enzymes are in the LU state with an intermolecular disulfide bond and with the active site Thr (Thr43 in StAhpC) also swung in comparison to the FF state. Our structural analysis of StAhpC and Prx4 mutants indicated that subtle modifications in the dimer-dimer interface may induce major changes in the dimer-decamer transitions at lower protein concentrations.

The presence of a hydroxyl group in the side chain is also important for the stabilization of decamers (compare Ser44 versus Ala44 and Thr44 versus Val44 in Fig. 3). This is likely related to the fact that the O atom in the side chain of Thr residues performs distinct polar interactions in the FF and LU states, in both cases possibly these interactions help to stabilize 2-Cys Prx in the decameric form (See Supplementary Fig. 11). In contrast, the few available structures of 2-Cys Prx in the dimeric form show that the active site Thr of Prx1^C83S^ from *R. norvegicus* possesses polar interactions distinct from the FF and LU decamers^20^. The hydroxyl group of the residue at position 44 is clearly important for the peroxidase activity (please compare the second order rate constants for Ser44 and Thr44 versus Ala44 and Val44, respectively in Table 1). Given the positive tendency between 2-Cys Prx decameric structure and activity, it is possible that Thr and certainly Ser residues stabilize the decamer, thereby allowing for optimal orientation of active site residues for peroxide reduction.

**Figure 4:**
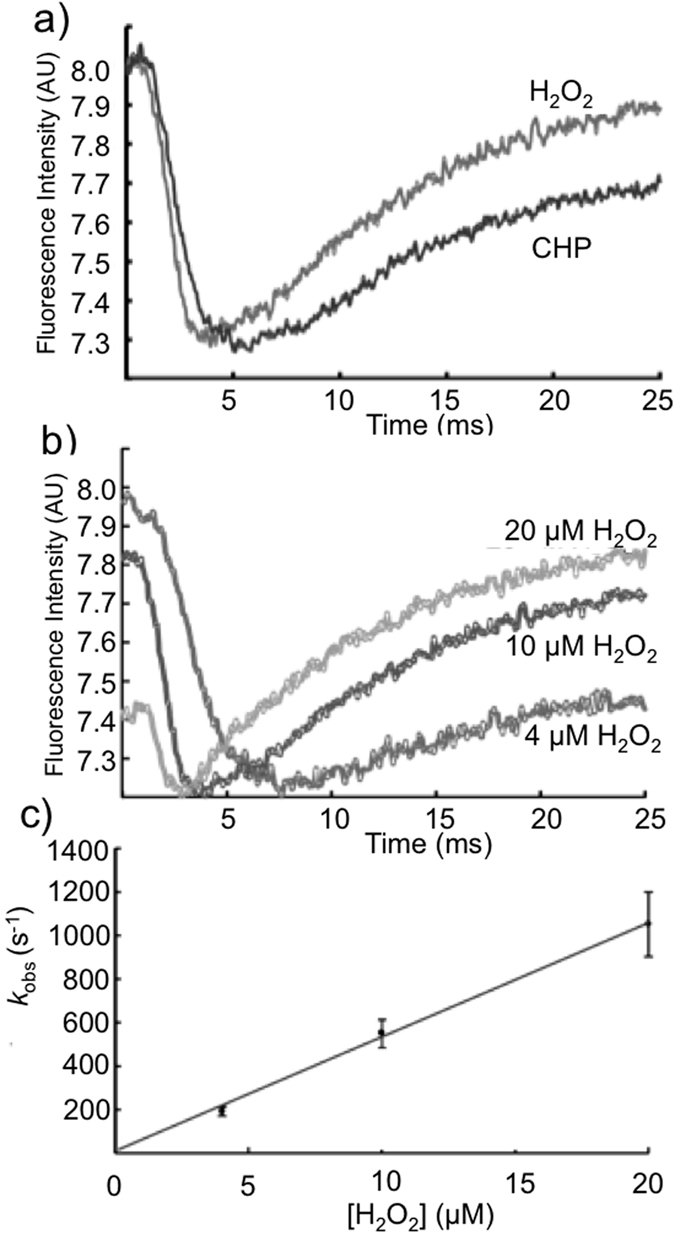
Hydroperoxide reduction by Tsa1 monitored by changes in intrinsic fluorescence. (**a**) Representative time course of Tsa1^WT^ (1 μM) oxidation by H_2_O_2_ or CHP (20 μM). (**b**) Oxidation of Tsa1^WT^ (1 μM) by increasing concentrations of H_2_O_2_ (4, 10 or 20 μM). Enzymes were previously reduced with 20 mM DTT for 30 minutes at room temperature, and excess DTT was removed by gel filtration. Total intrinsic fluorescence decays upon oxidation by hydroperoxides were followed (λ_ex_ = 280 nm; λ_em_ > 310 nm using a cutoff filter) at 25 °C. The assays were performed in 40 mM phosphate buffer (pH 7.4). (**c**) The *k*_*obs*_ values were obtained by fitting the stopped-flow data to single exponentials; second order rate constant was calculated from the slope of *k*_*obs*_ plot versus oxidant concentration.

**Figure 5:**
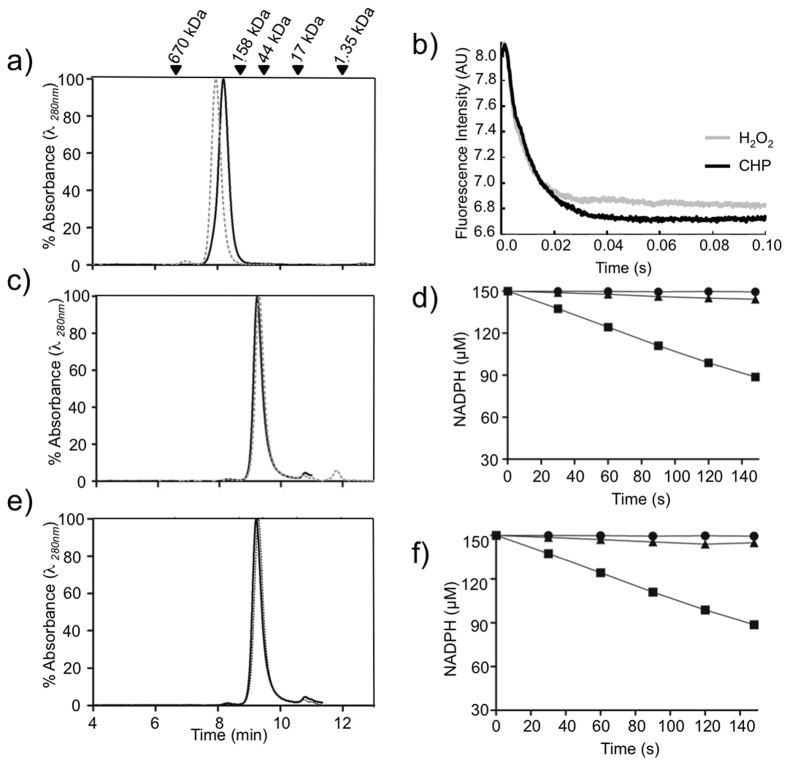
Structural and kinetic characterization of Tsa2, Tsa1^Y77A^ and Tsa1^S78D^. SEC elution profiles of reduced (solid) and oxidized (dashed) Tsa2 (**a**), Tsa1^Y77A^ (**c**) and Tsa1^S78D^ (**e**) at 50 μM concentration, as described in Fig. 3. (**b**) Oxidation of Tsa2 (1 μM) by H_2_O_2_ or CHP (20 μM) performed in 40 mM phosphate buffer (pH 7.4). Tsa2 was previously reduced with 20 mM DTT for 30 minutes at room temperature, and the excess DTT was removed by gel filtration. (**d**,**f**) Trx system coupled assays: the reaction mixtures contained 2-Cys Prx (1 μM), Trx1 (2 μM), TrxR1 (0.3 μM), NADPH (150 μM), in HEPES-NaOH 50 mM (pH 7.4) and sodium azide (1 mM) and DTPA (0.1 mM). The reactions were performed at 30 °C. Tsa1^Y77A^ (**d**) and Tsa1^S78D^ (**f**) are represented by (▲), whereas negative control without Prx and positive control with Tsa1^WT^ are represented by (●) and by (■), respectively.

However, these associations between decamer and activity end after C_P_ oxidation. For 2-Cys Prx containing Thr, this residue undergo a re-orientation during the FF-LU transition, which may disrupt the non-conventional CH-π hydrogen bond and perhaps various other neighboring interactions in the dimer-dimer interface, inducing decamer to dimer transition. In contrast, for 2-Cys Prx enzymes containing Ser, the FF-LU transition is smoother and the decameric oxidized structure is maintained throughout the catalytic cycle. It is tempting to speculate that the slow rise in fluorescence observed for the Tsa1^WT^ is associated with this decamer dissociation (Fig. 4a,b), since it was not observed for the Tsa1^T44S^ (See Supplementary Fig. 8) or for Tsa2 (Fig. 5b). If true, this would provide an opportunity to access the kinetics of decamer dissociation. However, at this moment, the mechanism behind of the slow rise in fluorescence during Tsa1 oxidation is still under investigation.

2-Cys Prx enzymes display complex structural and biochemical properties, involving distinct quaternary structures and oxidation states. Distinct post-translational modifications (hyperoxidation, glutathionylation and phosphorylation) in 2-Cys Prx also influence the quaternary structures they adopt and also their biological activities, indicating that mechanisms were positively selected for though evolution^26^,^27^,^28^. It is quite possible that these structural transitions among dimer, decamer and even higher molecular weight species relate to cellular functions, i.e. as peroxidase, chaperone, binding partner, enzyme activator and/or redox sensor (reviewed in ref. 18).

Our contribution here is to show that the active site Thr/Ser is also involved in modulating the stabilities of distinct 2-Cys Prx quaternary structures. The relationship between peroxidase activity and oligomerization is complex but disruption of dimer-dimer interactions in the reduced state is associated with a decreased catalytic efficiency with possible physiological significance as 2-Cys Prxs are considered the H_2_O_2_ sensors^7^,^8^,^10^.

## Material and Methods

### Site directed mutagenesis

The pET15b/*Tsa1* plasmid was used as template to generate the individual Tsa1 mutants carrying Thr44 substitutions to Ala (Tsa1^T44A^), Ser (Tsa1^T44S^) and Val (Tsa1^T44V^); Tyr77 to Ala (Tsa1^Y77A^) and Ser78 to Asp (Tsa1^S78D^). The mutagenesis protocols were performed according manufacturer instructions, using Quick Change II Kit (Stratagene) and the following primers: Tsa1^T44A^_F (5′ TGGCCTTCGCTTTCGTCTGT 3′), Tsa1^T44A^_R (5′ ACAGACGAAAGCGAAGGCCA 3′); Tsa1^T44S^_F (5′ TTGGCCTTCAGTTTCGTCTGT 3′), Tsa1^T44S^_R (5′ ACAGACGAAACTGAAGGCCAA 3′); Tsa1^T44V^_F (5′ TTGGCCTTCGTTTTCGTCTG 3′), Tsa1^T44V^_R (5′ CAGACGAAAACGAAGGCCAA 3′); Tsa1^S78D^_F (5′ TCCGAATACGACCTTTTGGCA 3′), Tsa1^S78D^_R (5′ TGCCAAAAGGTCGTATTCGGA 3′), Tsa1^Y77A^_F (5′ GACTCCGAAGCCTCCCTTTTG 3′), Tsa1^Y77A^_R (5′ CAAAAGGGAGGCTTCGGAGTC 3′). The reaction products were treated with *Dpn* I to remove the parental methylated plasmids and *E. coli* XL1-Blue strain was used as host in the transformations. Single colonies were selected and their plasmids extracted and sequenced with BigDye Terminator v3.1 Cycle Sequencing Kit using the automatic sequencer ABI 3730 DNA Analyser (Thermo Scientific) to confirm codon substitutions. The plasmids harboring the mutations were transformed in *E. coli* BL21 (*DE3*) strain by electroporation.

### Protein expression and purification

Single colonies of *E. coli* BL21 (*DE3*) strain containing the pET15b/*tsa1*, pET15b/*tsa1*^*T44A*^, pET15b/*tsa1*^*T44S*^, pET15b/*tsa1*^*T44V*^, pET15b/*tsa1*^*Y77A*^, pET15b/*tsa1*^*S78D*^ or pPROEx/*tsa2* plasmids were inoculated in LB medium (20 ml) containing 0.1 mg ampicillin/mL and grown overnight at 37 °C with shaking at 250 rpm. The following day the cultures were transferred to 1 L of fresh LB medium, and cultured further until OD_600_ reached 0.6–0.8. The overexpression of all the proteins was induced by the addition of 0.3 mM isopropyl-1-thio-β-D-galactopyranoside (IPTG) at 37 °C for 3hs with shaking at 250 rpm in an orbital shaker. Cells were harvested by centrifugation and cell pellets were resuspended in start buffer (50 mM sodium phosphate buffer, pH 7.4, 500 mM NaCl, 20 mM imidazole and 2 mM phenylmethyl sulfonyl fluoride - PMSF) and disrupted by sonication. The cell extracts were kept in ice during streptomycin sulfate (1%) treatment for 20 min. The supernatants were clarified by centrifugation, homogenized by filtration and purified by immobilized metal affinity chromatography (IMAC) using HisTrap column (GE Healthcare). Imidazole was removed by gel filtration using PD10 desalting column (GE Healthcare) and the purity of recombinant proteins was verified by SDS-PAGE.

### Protein quantification

All of the purified enzymes were quantified using the molar extinction coefficient for reduced *S. cerevisiae* Tsa1^WT^ obtained using the ProtParam tool (http://www.expasy.ch/tools/protparam.html) (ε_280_ = 23,950 M^−1^ cm^−1^), except for Tsa1Y77A for which molar extinction coefficient is different (ε_280_ = 22,460 M^−1^ cm^−1^).

### Protein reduction

Wild type and mutant proteins were initially reduced with 5 mM TCEP in 50 mM sodium phosphate buffer (pH 7.4), 50 mM NaCl, 100 μM diethylenetriaminepentaacetic acid (DTPA) and 1 mM sodium azide, for 30 minutes, at room temperature. In some cases, TCEP was replaced by DTT (20 mM). After reduction, excess of TCEP or DTT was removed using a PD10 column (GE Healthcare).

### Circular dichroism spectroscopy of Tsa1^WT^ and mutants carrying Thr44 substitutions

The CD spectra of Tsa1^WT^ and mutant proteins were obtained using a 0.1 cm path length cuvette containing 10 μM of protein sample in 10 mM Tris buffer (pH 7.4) and 100 mM NaF. The assays were carried out at 25 °C in a Jasco J-810 spectropolarimeter (Jasco Inc.). Spectra were presented as an average of eight scans recorded from 190 to 260 nm. The content of secondary structures in each protein was estimated using the CDNN 2.1 software^39^.

### Size-exclusion chromatography

Size-exclusion chromatography was performed by analytical HPLC (Jasco LC-2000 Plus) equipped with a PU 2880 Plus injector and a PDA MD 2018 detector (Jasco). The samples (50 μM in 100 mM Tris-HCl at pH 7.4) were separated by a system containing a Phenomenex BioSep-SEC-S3000 column (7.8 × 300 mm, 5 μm, resolution range of 15 to 2000 kDa, Phenomenex, Inc., Torrance, California, USA) using a flow rate of 1.0 mL/min in 100 mM Tris-HCl buffer (pH 7.4) and 50 mM NaCl. The elution profile was monitored by absorbance (λ = 280 nm). Bovine thyroglobulin (670 kDa), bovine gamma globulin (158 kDa), ovalbumin (44 kDa), myoglobin (17 kDa) and vitamin B_12_ (1.35 kDa) were used as molecular weight standards (Bio-Rad). The chromatograms were analyzed using Jasco BORWIN, version 1.50, software (Jasco). The enzymes were reduced or oxidized by treatment with 5 mM TCEP or 1.2 molar excess of H_2_O_2_, respectively, for 30 min at 25 °C.

### Determination of peroxidase activity using the Trx system coupled assay

Trx peroxidase activities were monitored by NADPH oxidation in a coupled assay^35^. *S. cerevisiae* Tsa1^WT^ and mutants (1.0 μM) were incubated with Trx1 (2 μM), TrxR1 (0.3 μM) and NADPH (150 μM) in 50 mM HEPES buffer (pH 7.4) containing sodium azide (1 mM) and DTPA (100 μM), at 30 °C. Reactions were initiated by addition of H_2_O_2_ or cumene hydroperoxide (CHP). All kinetic data were analyzed by non-linear regression using Michaelis-Menten equation^40^ (GraphPad Prism 5 software, GraphPad Software, Inc., San Diego).

### Kinetics of Tsa1 oxidation using the intrinsic fluorescence of the enzyme

Reduced Tsa1^WT^, Tsa1 mutants and Tsa2 enzymes (1 μM) in 40 mM sodium phosphate buffer (pH 7.4) were rapidly mixed with either H_2_O_2_ or CHP in excess in an Applied Photophysics SX-17MV stopped-flow spectrometer with a mixing time of approximately 2 ms. The reaction was followed by total fluorescence intensity decrease (λ_ex_ = 280 nm; λ_em_ > 310 nm using a cutoff filter) of the enzymes upon oxidation. Observed rate constants (*k*_*obs*_) were determined by fitting the stopped-flow data to single exponential functions. The apparent second order rate constants were determined from the slope of *k*_*obs*_ values plotted against hydroperoxide concentrations.

### Non-reducing SDS PAGE assays

To study reduction of 2-Cys Prx by DTT and Trx1, wild-type Tsa1 and mutant proteins were previously reduced as described before and treated with H_2_O_2_ at 1.2 molar ratio in 50 mM sodium phosphate buffer (pH 7.4) containing 50 mM NaCl, 100 μM DTPA and 1 mM sodium azide. Next, the enzymes were reduced by increasing concentrations of DTT (10 μM to 10 mM) or Trx (2.5 to 25 μM) during 1 minute. The reaction was stopped with denaturing buffer (62.5 mM Tris-HCl, pH 6.8, containing 4% SDS and 10% glycerol) together with alkylation by 50 mM N-ethylmaleimide (NEM). Oxidized Tsa1^WT^ (intermolecular disulfide) runs as a dimer in non-reducing 12% SDS-PAGE, whereas reduced Tsa1^WT^ (dithiol) runs as a monomer in the same conditions. In order to evaluate the disulfide formation in the Tsa1^WT^ and mutants, the proteins were initially reduced, as described above. The oxidation assays were performed using 10 μM of protein and 30 μM of H_2_O_2_, in 100 μL of final volume at room temperature, in 50 mM sodium phosphate buffer (pH 7.4) containing NaCl 50 mM, 100 μM DTPA and 1 mM sodium azide. The reactions were stopped with 10 μL of denaturing buffer and 50 mM NEM to alkylate the enzymes thiols, precluding formation unspecific disulfide bonds^41^ at the intervals of 0, 10, 30, 60, 90, 120, 300 and 600 seconds. Reactions were analyzed by non-reducing SDS-PAGE stained with Coomassie blue.

### Analysis of Prx1/AhpC crystallographic structures

Analysis of available crystallographic structures and figures were performed using Pymol (http://www.pymol.org) and Discovery Studio 4.0. Distances and angles of non-conventional hydrogen bonds (CH-π interactions) in Prx structures were determined using Discovery Studio 4.0 software (http://accelrys.com/products/discovery-studio)^32^.

## Additional Information

**How to cite this article**: Tairum, C. A. *et al*. Catalytic Thr or Ser Residue Modulates Structural Switches in 2-Cys Peroxiredoxin by Distinct Mechanisms. *Sci. Rep.* **6**, 33133; doi: 10.1038/srep33133 (2016).

## Supplementary Material

Supplementary Information

## Figures and Tables

**Table 1:** Correlation of the oligomeric state with the peroxidatic activity.

			Peroxidase Activity^b^		Apparent second order rate constant^c^	
	Oligomerization State^a^		Trx System (*k*_cat_/*K*_m_/M^−1^ s^−1^)		(M^−1^ s^−1^)	
	Reduced	Oxidized	H_2_O_2_	CHP	H_2_O_2_	CHP
Tsa1^WT^	Decamer	Dimer/Decamer	2.0 ± 0.3 × 10^4^	8.2 ± 1.1 × 10^4^	4.7 ± 2.2 × 10^7^	3.2 ± 0.7 × 10^6^
Tsa1^T44A^	Decamer	Dimer*/Decamer	8.8 ± 0.2 × 10^3^	2.2 ± 0.3 × 10^3^	3.8 ± 0.2 × 10^4^	7.6 ± 0.8 × 10^4^
Tsa1^T44S^	Decamer	Decamer	1.3 ± 0.2 × 10^4^	7.5 ± 0.9 × 10^4^	2.3 ± 0.3 × 10^7^	1.4 ± 0.3 × 10^7^
Tsa1^T44V^	Dimer*/Decamer	Dimer	7.3 ± 1.4 × 10^2^	0.0	ND	ND
Tsa1^Y77A^	Dimer	Dimer	0.0	0.0	ND	ND
Tsa1^S78D^	Dimer	Dimer	0.0	0.0	ND	ND
Tsa2^WT^	Decamer	Decamer	2.8 ± 0.7 × 10^4^	1.7 ± 0.9 × 10^5^	5.0 ± 1.7 × 10^6^	5.6 ± 0.4 × 10^6^

^*^Denote the predominant quaternary species; ND = Not Determined.

^a^Determined by size exclusion chromatography – [Prx] = 50 μM (see Fig. 3).

^b^Determined by the Trx system coupled assay – [Prx] = 1 μM (see supplementary Fig. 5).

^c^Determined by intrinsic fluorescence kinetics – [Prx] = 1 μM (see Fig. 4).
