# Supplementary material for: Catalytic Thr or Ser Residue Modulates Structural Switches in 2-Cys Peroxiredoxin by Distinct Mechanisms
Source: Sci Rep. 2016 Sep 15;6:33133. doi: 10.1038/srep33133 (PMC5024103; doi:10.1038/srep33133)
Supplement: Supplementary Information [file srep33133-s1.doc]

**Supplementary Information**

**CATALYTIC THR OR SER RESIDUE MODULATES STRUCTURAL SWITCHES IN 2-CYS PEROXIREDOXIN BY DISTINCT MECHANISMS.**

Carlos A. Tairum Jr^1^, Melina Cardoso Santos^1^, Carlos A. Breyer^1^, R. Ryan Geyer^2^, Cecilia J. Nieves^3^, Stephanie Portillo-Ledesma^3^, Gerardo Ferrer-Sueta^3^, José Carlos Toledo Jr.^2^, Marcos H. Toyama,^1^, Ohara Augusto^2^, Luis E. S. Netto^5*^& Marcos A. de Oliveira^1*^

^†^This work was supported by grants 07/50930-3 and 13/07937-8 from the Fundação de Amparo à Pesquisa do Estado de São Paulo

^1^ Instituto de Biociências, Campus do Litoral Paulista, Universidade Estadual Paulista Júlio de Mesquita Filho, São Vicente, São Paulo, Brazil, 11330-900.

^2^ Departamento de Bioquímica, Instituto de Química, Universidade de São Paulo, São Paulo, Brazil, 05508-090.

^3^ Facultad de Ciencias – Universidad de la República – Montevideo - Uruguay

^4^ Departamento de Química, Faculdade de Filosofia, Ciências e Letras de Ribeirão Preto da Universidade de São Paulo (FFCLRP – USP), 14040-90.

^5^ Departamento de Genética e Biologia Evolutiva, Instituto de Biociências, Universidade de São Paulo, São Paulo, Brazil, 05508-090.

^*^To whom correspondence should be addressed: Luis E. S. Netto, phone: 55-11-3091 7589, fax: 55-11-3091 7553, e-mail: nettoles@ib.usp.br and Marcos A. de Oliveira, phone: 55-13-3569 7148, fax: 55-13-3569 7146. e-mail: mao@clp.unesp.br.

**Supplementary Figures**


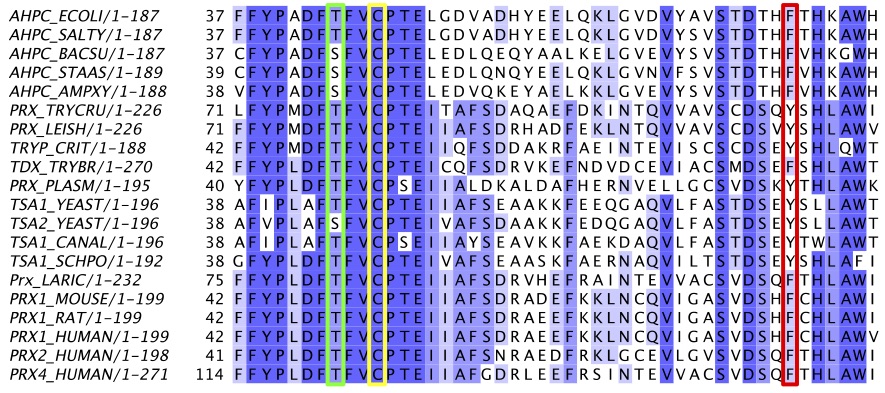


**Supplementary Figure 1. Sequence alignment of several 2-Cys Prx enzymes reveals conservation of catalytic Thr/Ser and corresponding Phe/Tyr involved in CH-π H bond.** Amino acid sequence alignment using Clustal Omega (Sievers *et al*., *Mol. Syst. Biol.* **7**, 539, 2011) and the figure generated by Jalview (Waterhouse *et al*., *Bioinformatics* **25**, 1189-91, 2009). Identical residues are shaded in blue and sequence similarity is indicated by a blue gradient based on the conservation of physicochemical characteristics. The species abbreviation initials are given in italics and are as follows: *Escherichia coli = AHPC_ECOLI* (NCBI accession number: P0AE08.2); *Salmonella typhimurium* = *AHPC**_SALTY* (P0A251.2); *Bacillus subtilis* = *AHPC_B**ACSU* (P80239.2); *Staphylococcus aureus* = *AHPC_STAAS* (Q6GC91.1); *Amphibacillus xylanus* = *AHPC_AMPXY* (BAA33808.1); *Trypanosoma cruzi* = *PRX_TRYCRU* (CAA06923.1); *Leishmania major* = *PRX_LEISH* (XP_001683326.1); *Crithidia fasciculata* = *TRYPCRIT* (AAC72300.1); *Trypanosoma brucei* = *TDX_TR**YBR* (Q26695.1); *Plasmodium vivax* = *PRX_PLASM* (XP_001616002.1); *Saccharomyces cerevisiae* = *TSA1_YEAST* (P34760.3) and *TSA2_YEAST* (Q04120.3); *Candida albicans* = *T**SA1_CANAL* (Q9Y7F0.1); *Schizosaccharomyces pombe* = *TSA1_SCHPO* (O74887.1); *Larimichthys crocea* = *PRX_LARIC* (377656258); *Mus musculus* = *PRX1_MOUSE* (P35700.1); *Rattus norvegicus* = *PRX1_RAT* (Q63716.1); *Homo sapiens* = *PRX1_HUMAN* (Q06830.1), *PRX2_HUMAN* (P32119.5) and *PRX4_HUMAN* (Q13162.1). The green box denotes the catalytic triad Thr/Ser, the red box the Tyr/Phe and the yellow box the C_P_. The fully conserved Arg (Arg123 in Tsa1) that also takes part in the catalytic triad is not represented here because it is far away in the primary sequence to the C_P_.

**
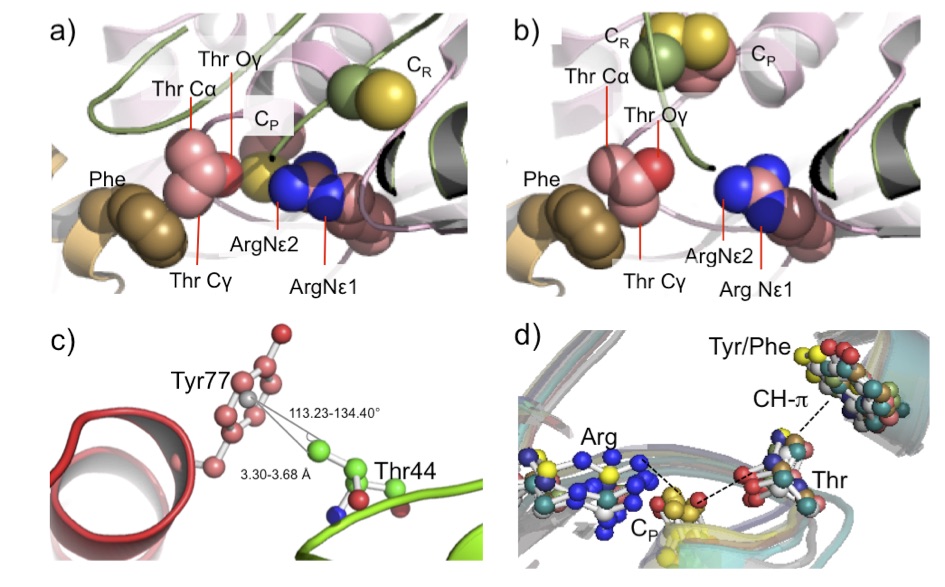
**

**Supplementary Figure 2. Structural features of CH-** **π H bond that is highly conserved in 2-Cys Prx in the FF state.** The structure of StAhpC, a 2-Cys Prx from *S. typhimurium* in the FF state (**a**; 4MA9) displays an intimate hydrogen bond network between Phe-Thr-C_P_-Arg which is lost upon disulfide formation in the LU state (**b**; 1YEP). The residues of catalytic triad and the conserved Tyr/Phe are represented by spheres and atoms are colored as follows: C = pink, gold, yellow or gray, according to the cartoon representation of the protein; N = blue; O = red; S = orange. (**c**) Diagram of Thr44 and Tyr77 from Tsa1 at the dimer-dimer interface, depicting the corresponding CH-π hydrogen bond. The distances/angles were determined using the program Discovery Studio 4.0 (Accelrys Software Inc., Discovery Studio Modeling Environment). (**d**) Overlap of the catalytic triad residues (C_P_ = C_p_, Arg and Thr) and CH-π hydrogen bonds between Thr and Phe/Tyr residues of several 2-Cys Prx crystallographic structures, all in the FF state and depicted in different colors as follow: *S. typhimurium* AhpC (yellow; 4MA9), *Leishmania major* PrxII (brown; 4K1F), *Schistosoma mansoni* Prx (white; 3ZTL), *Pseudosciaena crocea* Prx4 (cyan; 3QPM), *Homo sapiens* sulfinilate form of Prx2 (red; 1QMV), *Rattus norvegicus* Prx1^C51S^ (dark green; 2Z9S) and *S. cerevisiae* Tsa1^C47S^ (dark blue; 3SBC).


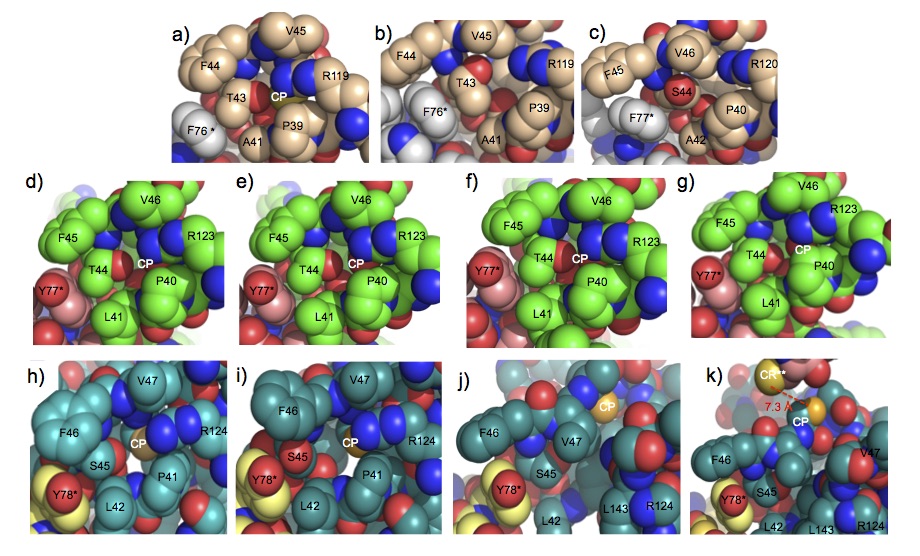


**Supplementary Figure 3. Environment around the active site Thr/Ser residue in oxidized and reduced states of the 2-Cys Prxs. (a,b)** StAhpC (with Thr in the catalytic triad) active site pocket environment represented in spheres in the reduced (**a**; 4MA9) and oxidized states (**b**; 1YEP). (**c**) Structure of *A. xylanus* 2-Cys Prx (with Ser in catalytic triad) in the oxidized state (**c**; 1WE0). (**a-c**) atoms are colored as follows: C = beige or white, N = blue, O = red, S = orange. (**d-g**) The Tsa1 structure (3SBC) in the FF state, evidencing similar conformations in the different monomers (A, B, G and H, respectively). The atoms in Tsa1^C47S^ structure are colored as follow: C = green or rose, N = blue, O = red. (**h-k**) Environment around active sites Ser residue (Ser45) in Tsa2 decameric structure (5DVB) (**h,i**). Both monomers are in the FF state, but in two different rotamers (monomers B and A, respectively). (**j,k**) Tsa2 monomers in the LU state (monomers E and J). The atoms in Tsa2^C48S^ structure are colored as follow: C = green or pale yellow, N = blue, O = red, S = orange. In all figures the asterisk (*) denotes residues form an adjacent dimer and double asterisk (**) denotes residues from the other subunit of the obligate dimer. The gamma atoms of the Ser48 (Cys48 in wild type protein) are colorized in orange for clearness.

**Supplementary Figure 4. CD spectra of native samples of Tsa1^WT^ and Thr44 mutants.** Spectra of the enzymes Tsa1^WT^ (blue), Tsa1^T44A^ (red), Tsa1^T44S^ (green) and Tsa1^T44V^ (purple). The protein concentration used in these experiments was 10 μM in 10 mM Tris buffer (pH 7.4) and 100 mM sodium fluoride. All spectra were recorded at 25°C and corrected against the buffer without protein. The graphical representations are averages from eight consecutive scans. The graphical representations of CD spectra from Tsa1 mutants were normalized based on the conversion of the maximum value of each spectrum to the maximum value obtained in the corresponding spectrum of Tsa1^WT^.

This experiment was performed to verify if the Thr44 amino acids substitutions provoked major perturbations on the overall Tsa1 secondary structure. All circular dichroism (CD) spectra indicated that the amino acid substitutions did not provoke major perturbations and were consistent with α-helix and β-sheet content of approximately 33% and 20%, respectively, which is in agreement with Tsa1 crystallographic data (Tairum *et al*., *J. Mol. Biol.* **424**, 28-41, 2012).

**Supplementary Figure 5. Steady state kinetic analysis of the peroxidase activities of Tsa1 and mutants by the Trx system coupled assay with increasing concentrations of H_2_O_2_ or cumene hydroperoxide (CHP).** Tsa1^WT^ (**a**), Tsa1^T44A^ (**b**), Tsa1^T44S^ (**c**) and Tsa1^T44V^ (**d**) were incubated with Trx system (Prx = 1 μM; Trx1 = 2 μM; TrxR1 = 0.3 μM; NADPH = 150 μM) in 50 mM HEPES (pH 7.4), 100 µM DTPA and 1 mM sodium azide at 30°C and variable concentrations of hydroperoxides as indicated on the x-axis. The reactions were monitored spectrophotometrically by the consumption of NADPH at 340 nm. In the insets, the reactions with CHP instead of H_2_O_2_ are shown under the same experimental conditions, except for (**d**), in which no NADPH consumption was observed. In this case, the reaction of 200 μM CHP with Tsa1^T44V^ (●) is shown together with a positive control (Tsa1^WT^ **-** ■) and a negative control (absence of enzyme **-** ▲). The initial rate values of each reaction were plotted against substrate concentration and the data were fit through a non-linear regression method using the Michaelis-Menten equation to obtain the kinetic parameters. (**e**) The determination of the dependence of the rate on Trx1 concentration by Tsa1^WT^. Trx concentrations used were: 0.1, 0.25, 0.5, 1.0, 2.0, 4.0, 6.0 and 8.0 μM. Tsa1 = 1 μM, TrxR = 0.3 μM, NADPH = 150 μM, H_2_O_2_ = 200 μM, HEPES 50 mM (pH 7.4), sodium azide = 1 mM and DTPA = 100 μM at 30°C. Similar results were obtained with the Tsa1 mutants for Thr44, indicating that Trx at 2 μM concentration does not limit catalysis.

**Supplementary Figure 6. Monomer-dimer conversion of Tsa1 and mutants as a function of the time after oxidation by H_2_O_2_ or CHP and reduction by DTT or Trx**. In this approach oxidized Tsa1^WT^ and Thr44 mutants (intermolecular disulfide) runs as a dimer in non-reducing 12% SDS-PAGE, whereas reduced Tsa1^WT^ and Thr44 mutants (dithiol) runs as a monomer in the same conditions. In all of the assays, Tsa1^WT^ (**a**), Tsa1^T44A^ (**b**), Tsa1^T44S^ (**c**), and Tsa1^T44V^ (**d**) at 10 μM concentration were previously reduced with 20 mM DTT for 30 minutes at room temperature and the DTT excess was removed by gel filtration. For the oxidation assays, the enzymes were treated with H_2_O_2_ or CHP (3 eq.). For the reduction assays, the enzymes were oxidized with H_2_O_2_ (1.2 eq.) and then reduced by DTT or Trx in concentrations denoted at the top of the figures. Assays were performed at room temperature in 10 mM HEPES (pH 7.4), 100 µM DTPA, 1 mM sodium azide. Reactions were stopped by the addition of buffer containing NEM (50 mM), 4% SDS, 10% glycerol, 62.5 mM Tris-HCl (pH 6.8) at the times indicated at the top of the figure. D = dimer, M = monomer and T – thioredoxin.

For the description of the results relative to Tsa1 oxidation by H_2_O_2_ and CHP, please see text in the main body.

We also investigated the effects of Thr44 substitutions on Tsa1 reduction by 1,4 dithiothreitol (DTT) or by Trx1. Pre-oxidized enzymes were treated with different amounts of DTT (1-100 eq) or Trx (1-25 eq) at short intervals. Reduction of Tsa1^WT^ and mutant proteins proceeded similarly, indicating that the residues at position 44 had a minor role in the reduction step.

We do not have a clear explanation for the appearance of more than one monomeric band that is more evident in the case of Tsa1^T44V^.

Chae *et al.*, (*Proc Natl Acad Sci U S A* **91**, 7022-6, 1994) also observed more than one monomeric band for Tsa1 that could not be explained by the loss of His tag. Possibly distinct bands correspond to monomers with different conformations

According to Chae *et al.* (1994): “whereas RC47S and RC170S each yielded at least one additional monomeric band (M2) just above Ml and one band each” Even for the non-recombinant Tsa1 protein purified from yeast more than one monomeric band was observed. The meaning of these bands and the effects of the mutations on their appearance waits further studies for elucidation.

**
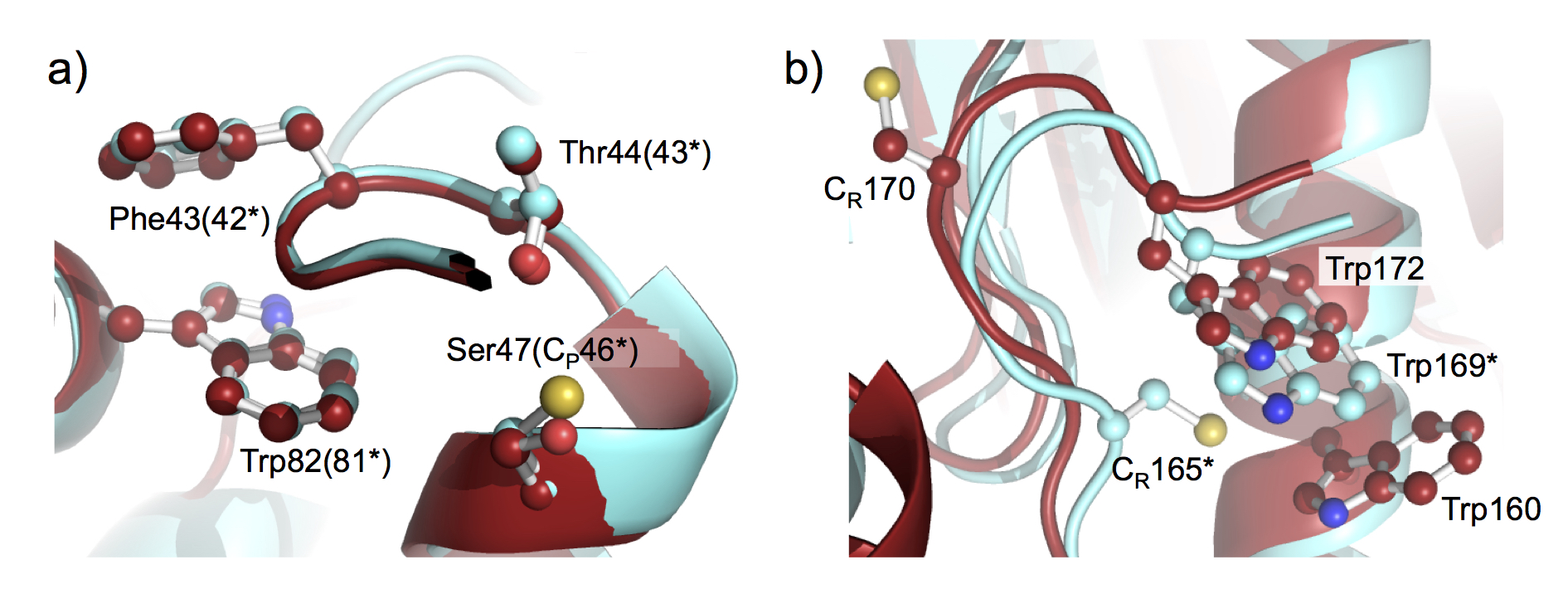
**

**Supplementary Figure 7. Structural comparison of Trp residues between Tsa1 and StAhpC in the vicinity of catalytic cysteines.** The Trp residues around C_P_ (or Ser substituting C_P_) (**a**) or C_R_ (**b**) are represented. Striking differences were found at Trp172 and C_R_ (170) in Tsa1 (3SBC, red), and at Trp169 and C_R_ (165) of StAhpC (4MA9, light blue). Furthermore, an additional Trp residue in Tsa1 is found close to C_R_. The residue numbering refers to Tsa1 residues and those numbers in parentheses with an asterisk are based on the numbering relative to StAhpC residues. The structures are represented in cartoon, while Trp and Cys residues are represented by balls and sticks. C = same color of the cartoon representations, N = blue, O = red, S = orange.

Positions of the Trp residues in close proximity to the C_R_ residues in Tsa1 and StAhpC are strikingly different.

**Supplementary Figure 8. Peroxide reduction by Tsa1 mutants**. Samples were previously reduced with 20 mM DTT by 30 minutes at room temperature and the excess was removed by gel filtration. In the reaction, 10 μM of H_2_O_2_ (red trace) or CHP (blue trace) was added to 1 μM of Tsa1^T44A^ (**a**), Tsa1^T44S^ (**b**) and Tsa1^T44V^ (**c**). Comparative traces decay of Tsa1^T44S^ with Tsa1^T44V^ (**d**). The assays were performed in phosphate buffer (40 mM) pH = 7.4 at 25 °C. The excitation wavelength was 280 nm and the emission was monitored in filter 310 nm cutoff. The values of *k* were obtained by single exponential curve.

**Supplementary Figure 9. Kinetic analysis of Tsa2, Tsa1^Y77A^ and Tsa1^S78D^.** The peroxidase activity of Tsa2 using H_2_O_2_ **(a)** and CHP **(b)** as substrates was evaluated by the Trx system coupled assay. Reaction mixtures contained: Tsa2 (1 μM), Trx1 (2 μM), TrxR1 (0.3 μM), NADPH (150 μM), in 50 mM HEPES-NaOH (pH 7.4), sodium azide (1 mM) and DTPA (100 μM), using variable concentrations of hydroperoxides. Experiments were performed as described in Supplementary Figure 5. Representative time courses of Tsa1^Y77A^ **(c)** and Tsa1^S78D^ **(d).**  Comparative fluorimetric traces (λ_ex_ = 280 nm; λ_em_ > 310 nm using a cutoff filter) of Tsa1^T44S^ with Tsa1^Y77A^ and with Tsa1^S78D^ (**e** and **f**, respectively). The enzymes (1 μM) previously reduced by DTT and then were oxidized with H_2_O_2_ or CHP (20 μM) in 40 mM phosphate buffer (pH 7.4) at 30°C. Non reducing SDS-PAGE assay for disulfide formation after H_2_O_2_ treatment. Tsa1^Y77A^ (**g**) and Tsa1^S78D^ (**h**) were pre-reduced, desalted and treated with H_2_O_2_ (30 μM of H_2_O_2_ for 10 μM of enzyme). The experiments were performed at room temperature in 10 mM HEPES (pH 7.4), 100 µM DTPA, 1 mM sodium azide. Reactions were stopped by addition of buffer containing 50 mM NEM, 4% SDS, 10% glycerol, 62.5 mM Tris-HCl (pH 6.8) at the times indicated at the top of the figure. D = dimer; M = monomer.

**
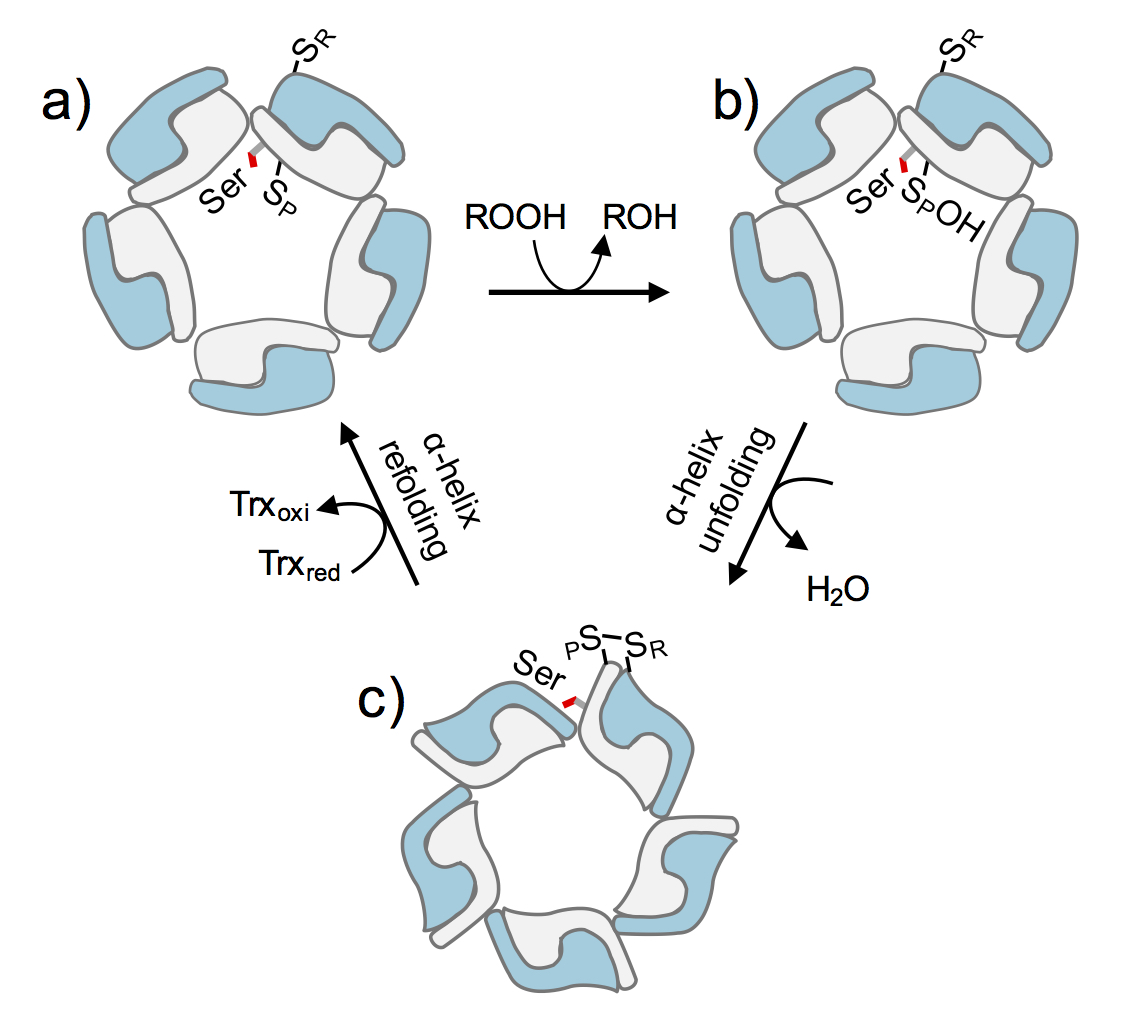
**

**Supplementary Figure 10. Proposed mechanism for 2-Cys Prx (AhpC-Prx1) reduction of hydroperoxides** **with a Ser in the catalytic triad**. **(a)** C_P_ in the fully folded (FF) form (S^-^p) displays high reactivity towards hydroperoxides and stabilizes the decamer state. This quaternary structure corresponds to a strong decamer. **(b)** The oxidation product (sulfenic acid - SpOH), also a strong decamer, can have two outcomes, condensation or hyperoxidation. For simplicity, the hyperoxidation shunt was omitted here. **(c)** The intermolecular disulfide is formed after a local unfolding process, since the two Cys residues are far away in the FF state. In contrast to 2-Cys Prx enzymes with Thr, the shorter side chain of Ser does not destabilize the decamer. The reduced form of typical 2-Cys Prx, which favors the strong decamer, is regenerated by Trx.

**Supplementary Figure 11. Interactions of active site Thr/Ser with main chain residues in the FF and LU states.** Polar interactions of the Thr Oγ with the main chain amino (Phe) or carbonyl group (Ala/Leu) of StAhpC (**a,b**), *R. norvegicus* Prx1 (**c,d**), human Prx4 (**e,f**) in the FF (reduced; **a,c,e**) or LU (oxidized; **b,d,f**) states. As a consequence of the FF-LU transition, the distances between the polar interactions increase considerably. The greatest distances between polar interactions in the LU state are from *R. norvegicus* Prx1 (**d**). The carbon atoms from each monomer are represented by different colors: StAhpC = blue and white; Prx1 = green and white; Prx4 = yellow and white. The remaining atoms are colored as follows: N = blue, O = red, S = orange. Red dashed line denotes the distance between the atoms (in angstroms) participating in polar interactions. The black dashed line denotes the CH-π bond. MC denotes main chain. PDB code and oxidation state is provided at the bottom right corner of each panel.

**Supplementary Table 1. Crystallographic structures of the 2-Cys Prx enzymes analyzed in this work.**

| PDB code | Protein | Organism | Redox state | Quaternary Structure |
| --- | --- | --- | --- | --- |
| 1WE0^1^ | AhpC | *Amphibacillus xylanus* | Oxidized (LU) | Decamer |
| 1QMV^2^ | Prx2 | *Homo sapiens* | Reduced (FF) | Decamer |
| 3TKP^3^ | Prx4 | *H. sapiens* | Reduced (FF) | Decamer |
| 3TKR^3^ | Prx4 | *H. sapiens* | Reduced/Oxidized (FF/LU) | Decamer |
| 3TJG^3^ | Prx4 | *H. sapiens* | Oxidized (LU) | Decamer |
| 3QPM^4^ | Prx4 | *Larimichthys crocea* | Reduced (FF) | Decamer |
| 4K1F^5^ | Tryp3 | *Leishmania major* | Reduced (FF) | Decamer |
| 2I81^6^ | Prx | *Plasmodium vivax* | Reduced (FF) | Decamer |
| 2Z9S^7^ | HBP23 | *Rattus norvegicus* | Reduced (FF) | Decamer |
| 1QQ2^8^ | Prdx1 | *Rattus norvegicus* | Oxidized (LU) | Dimer |
| 3SBC^9^ | Tsa1 | *S. cerevisiae* | Reduced (LU) | Decamer |
| 5DVB^10^ | Tsa2 | *S. cerevisiae* | Reduced/oxidized (FF/LU) | Decamer |
| 4MA9^11^ | AhpC | *Salmonella typhimurium* | Reduced (FF) | Decamer |
| 1N8J^12^ | AhpC | *S. typhimurium* | Reduced (FF) | Decamer |
| 1YF0^13^ | AhpC | *S. typhimurium* | Oxidized (LU) | Decamer |
| 1YF1^13^ | AhpC | *S. typhimurium* | Oxidized (LU) | Decamer |
| 1YEP^13^ | AhpC | *S. typhimurium* | Oxidized (LU) | Decamer |
| 1YEX^13^ | AhpC | *S. typhimurium* | Oxidized (LU) | Decamer |
| 3ZTL^14^ | SmPrxI | *Schistosoma mansoni* | Reduced (FF) | Decamer |

**1)** Kitano *et al.*, *Proteins*. 59, 644–647 (2005); **2)** Schröder *et al.*, *Structure*. 8, 605–615 (2000); **3)** Wang *et al.*, *Biochem. J.* 441, 113–118 (2012); **4)** Mu *et al.*, *PLoS One*. 8, e57061 (2013); **5)** Brindisi *et al.*, *Sci. Rep*. 5, 9705 (2015); **6**) Artz, J.D. *et al*. *To be published*; **7)** Matsumura *et al*., *J. Biol. Chem*. 283, 284 –293 (2008); **8)** Hirotsu *et al.*, *Proc. Natl. Acad. Sci.* 96, 12333–12338 (1999); **9)** Tairum *et al.*, *J Mol. Biol.* 424, 28–41 (2012); **10)** Nielsen *et al*., *Acta Crystallogr. Sect. D Struct. Biol.* 72, 158–167 (2016); **11)** Perkins *et al*., *Biochemistry*. 52, 8708–21 (2013); **12**) Wood, Z.A., Poole, L.B., Karplus, P.A. (2003) *Science* 300: 650 –653; **13)** Parsonage *et al.*, *Biochemistry*. 44, 10583–10592 (2005); **14)** Saccoccia *et al.*, *Structure*. 20, 429–439 (2012).

**Supplementary Table 2. Apparent Michaelis-Menten parameters for Tsa1^WT^ and mutants.** Mono-substrate approach was employed by varying hydroperoxide concentrations. Rates were followed by NADPH oxidation. Tsa1^WT^ or mutants (1.0 μM), Trx1 (2μM), TrxR1 (0.3 μM), NADPH (150 μM) and H_2_O_2_ or CHP. The experiments were performed at 30°C. The results represent three independent experiments for each enzyme, according to data described on supplementary figure 5.

|  | **H_2_O_2_** | | | **CHP** | | |
| --- | --- | --- | --- | --- | --- | --- |
|  | K_M_ (µM) | *k*_cat_ (s^-1^) | *k*_cat_/K_M_ (M^-1^s^-1^) | K_M_ (µM) | *k*_cat_ (s^-1^) | *k*_cat_/K_M_ (M^-1^s^-1^) |
| Tsa1^WT^ | 28 ± 5 | 0.60 ± 0.02 | 2.0 ± 0.3 × 10^4^ | 8.80 ± 0.05 | 0.7 ± 0.1 | 8.2 ± 1.1 × 10^4^ |
| Tsa1^T44A^ | 80 ± 10 | 0.70 ± 0.03 | 8.8 ± 0.2 × 10^3^ | 300 ± 100 | 0.7 ± 0.1 | 2.2 ± 0.3 × 10^3^ |
| Tsa1^T44S^ | 53 ± 9 | 0.70 ± 0.03 | 1.3 ± 0.1 × 10^4^ | 9.9 ± 1.2 | 0.7 ± 0.1 | 7.5 ± 0.9 × 10^4^ |
| Tsa1^T44V^ | 520 ± 80 | 0.40 ± 0.01 | 7.3 ± 1.4 × 10^2^ | - | - | - |

Supplementary Text

Mutations of catalytic Arg123 (Tsa1^R123G^ and Tsa1^R123K^) in Tsa1 were also generated and characterized using similar approaches as those described throughout the manuscript for residue Thr44. The main features about these mutant proteins were (data not shown):

1. Tsa1^R123G^ and Tsa1^R123K^ presented quaternary structures quite similar to Tsa1^WT^.
2. Peroxidase activities were evaluated by Trx system coupled assay and by the disulfide formation in non-reducing SDS-PAGE. Tsa1^R123G^ and Tsa1^R123K^ both displayed only residual activity regardless of the assay system employed, which is in agreement with previous reports (Flohé, L. *et al*., *Arch. Biochem. Biophys.* **397,** 324–335, 2002; Montemartini, M. *et al*., *Eur. J. Biochem. FEBS* **264,** 516–524, 1999; Nagy, P. *et al.*, *J. Biol. Chem.* **286,** 18048–18055, 2011; Portillo-Ledesma, S. *et al.*, *Biochemistry* **53,** 6113–6125, 2014).
